# Microbial production of vitamin B_12_: a review and future perspectives

**DOI:** 10.1186/s12934-017-0631-y

**Published:** 2017-01-30

**Authors:** Huan Fang, Jie Kang, Dawei Zhang

**Affiliations:** 1 Tianjin Institute of Industrial Biotechnology, Chinese Academy of Sciences, Tianjin, 300308 China; 2 Key Laboratory of Systems Microbial Biotechnology, Chinese Academy of Sciences, Tianjin, 300308 China; 3 University of Chinese Academy of Sciences, Beijing, 100049 China; 4 College of Biotechnology and Food Science, Tianjin University of Commerce, Tianjin, 300134 China

**Keywords:** Vitamin B_12_, Biosynthesis, Metabolic regulation, Synthetic biology, *Escherichia coli*, Metabolic engineering

## Abstract

Vitamin B_12_ is an essential vitamin that is widely used in medical and food industries. Vitamin B_12_ biosynthesis is confined to few bacteria and archaea, and as such its production relies on microbial fermentation. Rational strain engineering is dependent on efficient genetic tools and a detailed knowledge of metabolic pathways, regulation of which can be applied to improve product yield. Recent advances in synthetic biology and metabolic engineering have been used to efficiently construct many microbial chemical factories. Many published reviews have probed the vitamin B_12_ biosynthetic pathway. To maximize the potential of microbes for vitamin B_12_ production, new strategies and tools are required. In this review, we provide a comprehensive understanding of advances in the microbial production of vitamin B_12_, with a particular focus on establishing a heterologous host for the vitamin B_12_ production, as well as on strategies and tools that have been applied to increase microbial cobalamin production. Several worthy strategies employed for other products are also included.

## Background

Vitamin B_12_, also known as cyanocobalamin, belongs to the cobalamin family of compounds, which are composed of a corrinoid ring and an upper and lower ligand. The upper ligand can be adenosine, methyl, hydroxy, or a cyano group [1]. Vitamin B_12_ is synthesized by prokaryotes and inhibits the development of pernicious anemia in animals.

Microbial de novo biosynthesis of vitamin B_12_ occurs through two alternative routes: the aerobic or anaerobic pathway, in bacteria and archaea, respectively. Some strains can also synthesize cobalamin by absorbing corrinoids via a salvage pathway, as shown in Table 1. Tetrapyrrole compounds including cobalamin, heme, and bacteriochlorophyll, are derived from δ-aminolevulinate (ALA) and a complex interdependent and interactional relationship exists among these tetrapyrrole compounds in numerous bacterial species [2]. To maintain vitamin B_12_ at stable levels, its biosynthesis and transportation is regulated by a cobalamin riboswitch in the 5′ untranslated regions (UTR) of the corresponding genes.

**Table 1:** Cobalamin biosynthetic pathway in microbes

Microorganisms	De novo synthesis pathway	Salvage pathway	References
Aerobes			
*Pseudomonas dentrificans*	Yes	Yes	[3]
*Rhodobacter capusulatus*	Yes	Yes	[3]
*Rhodobacter sphaeroides*	Yes	Yes	[3]
*Sinorhizobium meliloti*	Yes	Yes	[3]
Anaerobes			
*Salmonella typhimurium*	Yes	Yes	[4]
*Bacillus megaterium*	Yes	*	[5]
*Propionibacterium shermanii*	Yes	*	[5]
*Escherichia coli*	No	Yes	[4]
*Thermotoga* sp. RQ2	No	No	[6]
*Thermotoga maritima* MSB8	No	No	[6]
*Thermotoga neapolitana*	No	No	[6]
*Thermotoga petrophila*	No	No	[6]
*Thermotoga naphthophila*	No	No	[6]
*Thermotoga thermarum*	No	Yes	[6]
*Thermotoga lettingae*	No	Yes	[6]
*Fervidobacterium nodosum*	No	Yes	[6]
*Thermosipho melanesiensis*	Yes	Yes	[6]
*Thermosipho africanus*	Yes	Yes	[6]
*Kosmotoga olearia*	No	Yes	[6]
*Mesotoga prima*	No	No	[6]
*Petrotoga mobilis*	No	No	[6]

Unidentified pathways are marked with “*”

Large scale industrial production of vitamin B_12_ occurs via microbial fermentation, predominantly utilizing *Pseudomonas denitrificans*, *Propionibacterium shermanii*, or *Sinorhizobium meliloti* [7]. However, these strains have several shortcomings, such as long fermentation cycles, complex and expensive media requirements, and a lack of suitable genetic systems for strain engineering. To date, most of the research on these producers has focused on traditional strategies, such as random mutagenesis and fermentation process optimization, with only limited research on metabolic engineering. Recently, engineers have shifted their attention to *Escherichia coli* as a platform for vitamin B_12_ production. *E. coli* has become a well-studied cell factory that has been extensively used for the production of various chemicals, such as terpenoids, non-natural alcohols, and poly-(lactate-co-glycolate) [8–10]. Furthermore, metabolic engineering and synthetic biology strategies have been extensively applied to improve the production of these compounds [11, 12]. *Escherichia coli* synthesizes ALA via the C_5_ pathway and has been used as a microbial cell factory to produce ALA via C_4_ and C_5_ pathways [13, 14] and *E. coli* can also synthesize vitamin B_12_ via the salvage pathway. The closely related *Salmonella typhimurium* is able to synthesize vitamin B_12_ de novo. Many genes involved in vitamin B_12_ biosynthesis in *S*. *typhimurium* have been shown to be functional in *E. coli* [15–17]. Transfer of 20 genes from the *S*. *typhimurium cob* locus allowed the production of vitamin B_12_ in *E. coli* [18]. These advantages facilitate the de novo production of vitamin B_12_ in *E. coli*.

Due to the complexity of the pathway and its metabolic regulation, numerous studies have been performed by various groups on the vitamin B_12_ biosynthesis. This review provides an overview of the vitamin B_12_ biosynthesis and its metabolic regulation, along with an investigation of several strategies for the development of microbial cell factories, including synthetic biology and metabolic engineering, among others. Since research on the engineering of vitamin B_12_ producing strains remains limited, related strategies for other chemicals are also outlined.

## Cobalamin biosynthetic pathway

### De novo pathway

**Fig. 1:**
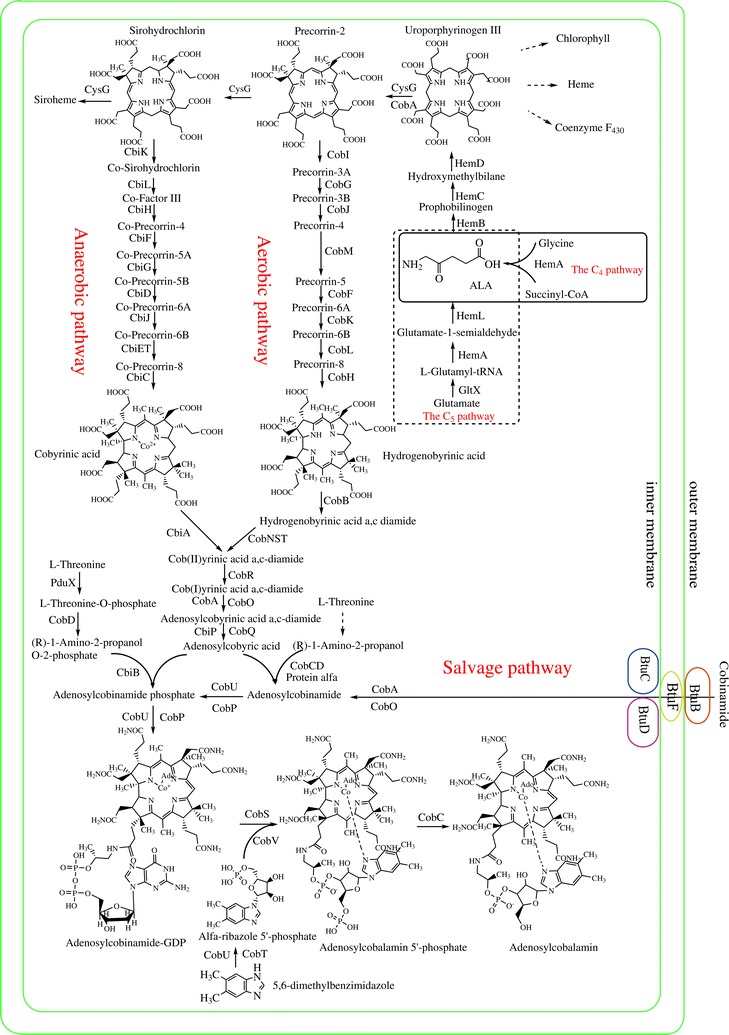
Biosynthetic pathways of tetrapyrrole compounds. ALA is synthesized by either the C_4_ or the C_5_ pathway. Adenosylcobalamin is synthesized via the de novo or via salvage pathways. The enzymes shown in the adenosylcobalamin biosynthetic pathway originate from *P. denitrificans or S. typhimurium,* which either use the aerobic pathway or the anaerobic pathway, respectively

As mentioned above, cobalamin can be synthesized de novo in prokaryotes through two alternative routes according to the timing of cobalt insertion and the molecular oxygen requirement. These pathways are the aerobic pathway, which has been best studied in *P. denitrificans*, and the anaerobic pathway, which has been best studied in *S. typhimurium*, *Bacillus megaterium*, and *P. shermanii*, [19]. Both routes differ in terms of cobalt chelation (via hydrogenobyrinic a, c-diamide with the CobNST complex in the aerobic pathway, and via precorrin-2 with CbiK in *S. typhimurium*) and oxygen requirements (the aerobic pathway requires oxygen to promote ring-contraction, while the anaerobic pathway does not require oxygen in this step) (Fig. 1).

Biosynthetic pathways of tetrapyrrole compounds: ALA is synthesized by either C_4_ or C_5_ pathway and adenosylcobalamin is synthesized either via de novo or salvage pathways. The enzymes shown in the adenosylcobalamin biosynthetic pathway originate from *P. denitrificans or S. typhimurium,* which use either the aerobic pathway or anaerobic pathway, respectively.

The first committed precursor of the tetrapyrrole synthesis pathway is ALA. ALA is synthesized by either the C_4_ pathway or the C_5_ pathway. In the C_4_ pathway, the enzyme ALA synthase from glycine and succinyl-CoA catalyzes the formation of ALA. In the C_5_ pathway, ALA is synthesized from glutamate through three enzymatic reactions [20]. Two molecules of ALA are condensed to form monopyrrole porphobilinogen by porphobilinogen synthase and four porphobilinogen molecules are then polymerized and cyclized to form uroporphyrinogen III. This reaction is catalyzed by the enzymes porphobilinogen deaminase and uroporphyrinogen III synthase. Methylation of uroporphyrinogen III at C-2 and C-7 results in the synthesis of precorrin-2 (which is a common precursor of cobalamin), siroheme, and coenzyme F_430_ [7, 21]. In *P. denitrificans*, CobA catalyzes this methylation step. In *S. typhimurium* and *E. coli*, the fusion enzyme CysG is shared between siroheme and cobalamin synthesis. The N-terminus of CysG has dehydrogenase/ferrochelatase activity and the C-terminus has uroporphyrinogen III methyltransferase activity. In *S. cerevisiae,* MET1p functions as a uroporphyrinogen III methyltransferase [22].

The aerobic and anaerobic pathways diverge at precorrin-2 and converge at coby(II)rinic acid a, c-diamide. Eight peripheral methylation reactions occur during de novo cobalamin biosynthesis, within identical temporal and spatial orders in both the aerobic and anaerobic pathways. Many of the methyltransferase enzymes involved in these reactions show high degrees of sequence similarity [23].

Cob(I)yrinic acid a,c-diamide is adenosylated to form adenosyl cobyrinic acid a,c-diamide. Cob(I)yrinic acid a,c-diamide adenosyltransferase can also adenosylate other corrinoids, where at least the a and c positions of the carboxyl groups are amidated. Adenosyl cobyrinic acid a,c-diamide is subjected to four stepwise amidation reactions at carboxyl groups at positions b, d, e, and g to yield adenosyl cobyric acid. Two separate methods have evolved to attach (*R*)-1-amino-2-propanol or (*R*)-1-amino-2-propanol phosphate at the f position of the carboxyl group of adenosyl cobyric acid in the aerobic and anaerobic pathways. In the anaerobic pathway, the linker between the corrinoid ring and the lower axial ligand is phosphorylated prior to attachment of the corrinoid ring. The enzyme PduX from *S. enterica* is an l-threonine kinase used in the de novo synthesis of coenzyme B_12_; however, it is not involved in the cobinamide salvage pathway [17]. l-threonine O-3-phosphate is then decarboxylated to yield (R)-1-amino-2-propanol O-2-phosphate via CobD in *S. typhimurium* LT2 [15]. However, in *P. denitrificans* it is most likely (although proof remains to be published), that (*R*)-*1*-amino-2-propanol is directly attached to the corrinoid ring via protein α and β. Currently, protein α remains as yet unknown and protein β is a complex of CobC and CobD. The molecule is then phosphorylated by CobP, which is a bifunctional enzyme possessing ATP:AdoCbi (adenosylcobinamide) kinase and GTP:AdoCbi-P guanylyltransferase activity [24]. Two additional reactions transfer lower axial ligands onto AdoCbi-GDP, thus producing adenosylcobalamin (AdoCbl). There are two alternative views of the last step in vitamin B_12_ biosynthesis. One view is that the last reaction in the biosynthesis of AdoCbl involves the addition of α-ribazole, catalyzed via cobalamine synthase. However, in *S. typhimurium*, α-ribazole 5′-phosphate is added to AdoCbi-GDP and thus, the last reaction would be the dephosphorylation of AdoCbl 5′-phosphate to AdoCbl, catalyzed by an AdoCbl-5-P phosphatase (CobC) [25]. The nucleotide loop assembly pathway is the last characterized reaction in the vitamin B_12_ biosynthesis. BluB from *S. meliloti* is a member of the reduced form of nicotinamide-adenine dinucleotide (NADH)/flavin mononucleotide (FMN)-dependent nitroreductase family, which can convert FMNH_2_ to DMB (5, 6-dimethylbenzimidazole) [26, 27]. In the anaerobic bacterium *E. limosum*, 5-aminoimidazole ribotide is converted to DMB by enzymes encoded in the *bza* operon [28] and subsequently, CobT can activate a range of lower ligand substrates including DMB, which determine cobamide diversity [29].

### Salvage pathway

The salvage pathway is a cost-effective way (in terms of energy) for bacteria and archaea to obtain cobalamin. In gram-negative bacteria, exogenous corrinoids are transported into the cell via an ATP-binding cassette (ABC) transport system, consisting of BtuC, BtuD, and BtuF, which are membrane permease, ATPase, and periplasmic-binding protein components, respectively. BtuB is a TonB-dependent transporter located in the outer membrane, delivering corrinoid to the periplasmic corrinoid-binding protein BtuF. The latter then delivers corrinoid to the BtuCD complex located in the inner membrane [30]. Archaea also use ABC transporters for corrinoid uptake. Archaeal orthologs of the bacterial BtuC, BtuD, and BtuF have been found in *Halobacterium* sp. strain NRC-1 [31]. Subsequent to transport through the membrane, cobinamide is adenosylated by ATP:co(I)rrinoid adenosyltransferases (ACATs). Three families of ACATs exist, namely: CobA, EutT, and PduO [32]. In bacteria, AdoCbi is the substrate for a bifunctional enzyme (CobU in *S. typhimurium* or CobP in *P. denitrificans*) with kinase and guanylyltransferase activities. In archaea, the *cbiZ* gene encodes an amidohydrolase that converts AdoCbi to adenosylcobyric acid, which is condensed with 1-aminopropanol-O-2-phosphate by an AdoCbi-P synthase (CbiB) to yield AdoCbi-P. Since the archaeal enzyme lacks AdoCbi kinase activity, CobY, which has GTP:AdoCbi-P guanylyltransferase activity, is used to transfer guanylyl to AdoCbi-P [30, 33]. Identical to the de novo pathway, two additional reactions transfer lower axial ligands onto AdoCbi-GDP to produce AdoCbl in the salvage pathway.

## Metabolic regulation of cobalamin synthesis

### The relationship between cobalamin, heme, and chlorophyll

Photosynthetic bacteria possess all three tetrapyrrole compounds including cobalamin, heme, and bacteriochlorophyll (Fig. 1), which share the same biosynthetic pathway from ALA to uroporphyrinogen III. Excess heme inhibits HemA via feedback inhibition, reducing the flux of cobalamin and bacteriochlorophyll, while heme limitation weakly regulates *hemCD*, *hemH*, and *hemA* expression in *E. coli* [34]. However, not just a competitive relationship exists among the tetrapyrrole compounds, as these compounds also share a complex interdependent and interactional relationship [2]. Hydrogenobyrinic acid synthase from *Rhodobacter capsulatus* possesses two Fe-S centers, a flavin and a heme group [35]. *Rhodobacter capsulatus* requires a cobalamin cofactor to form the isocyclic ring of chlorophyll [36]. Synthesis of heme is also cobalamin dependent, as heme synthesis requires S-adenosylmethionine as a methyl group donor, while S-adenosylmethionine synthesis involves a cobalamin-dependent enzyme [37]. This complex relationship is also manifest at the transcriptional level. E.g., cobalamin participates in the regulation of bacteriochlorophyll synthesis via the AerR-CrtJ regulatory pair in *R. capsulatus.* CrtJ is responsible for the repression of bacteriochlorophyll during aerobic growth [38], while AerR functions as an anti-repressor of CrtJ, inhibiting CrtJ binding to the *bchC* promoter when cobalamin is bound to the conserved histidine (His145) of AerR [39]. In *R. sphaeroides*, heme affects the ability of PpsR to regulate genes that are involved in the tetrapyrrole biosynthesis by binding PpsR and changing its DNA binding pattern. Thus, PpsR functions as both a redox and a heme sensor to coordinate cellular heme and bacteriochlorophyll levels [38]. This complex relationship between tetrapyrrole compounds is a consequence of natural evolution.

### Regulation of the key enzyme S-Adenosyl-l-methionine: uroporphyrinogen III methyltransferase (SUMT)

Feedback inhibition of a key enzyme located at the branch point of a biosynthetic pathway is a common method for metabolic regulation in microbes. In many microorganisms, SUMT regulates cobalamin flux. In *P. denitrificans*, SUMT is sensitive to feedback inhibition by cobalamin and corrinoid intermediates, and exhibits substrate inhibition at uroporphyrinogen III concentrations above 2 µM [40]. *Bacillus megaterium* SUMT exhibits substrate inhibition at uroporphyrinogen concentrations above 0.5 µM [41], while *P. denitrificans* SUMT is inhibited by uroporphyrinogen concentrations above 0.2 µM [40]. Fortunately, uroporphyrinogen III methyltransferase shows limited substrate inhibition. As an example, the uroporphyrinogen III methyltransferase of *Methanobacterium ivanovii* and *Paracoccus pantotrophus* were not inhibited by uroporphyrinogen III, even at concentrations of up to 20 µM [42, 43]. The identification of new SUMT enzymes that are insensitive to feedback inhibition to replace the native enzyme of industrial strains may be an effective method to improve vitamin B_12_ yield.

### Cobalamin riboswitches

The cobalamin riboswitch is the predominant form of metabolic regulation to control vitamin B_12_ concentration in microbes. Riboswitches are evolutionarily conserved non-coding regions that are situated in the 5′-untranslated region of mRNAs that regulate gene expression in response to direct binding of intracellular metabolites by the RNA itself [44]. Riboswitches are composed of two functional domains: one domain serves as an evolutionarily conserved natural aptamer, binding the target metabolite with high selectivity and affinity, while the other domain harnesses allosteric changes in RNA structure, caused by aptamer-ligand complex formation, to control expression of an adjacent gene or operon. Johnson et al. solved the crystal structures of two different classes of cobalamin riboswitches, which share a common four-way junction (P3–P6 helices), forming the core receptor domain that is responsible for cobalamin binding, but use distinct peripheral extensions (P8–P11) to recognize different cobalamin derivatives [45]. The P6 extension is present for the AdoCbl binding class, while it is absent for the methylcobalamin and the aquocobalamin binding classes [45–47]. A kissing-loop interaction between loop L5 of the cobalamin-binding core and L13 of the regulatory domain regulates the expression machinery [46]. Holmstrom et al. identified the conformational mechanism responsible for the regulation of gene expression by a hydroxocobalamin binding riboswitch (*env8*HyCbl). The authors utilized single-molecule fluorescence resonance energy transfer techniques [48]. Binding of hydroxocobalamin promotes the formation of the L5–L13 kissing-loop, which sequesters the Shine-Dalgarno sequence via base pairing, thus preventing translation initiation. Cobalamin riboswitches participate in the regulation of cobalamin biosynthesis and transport at the transcriptional or translational levels, such as the *btuB* gene of *E. coli* and the *cob* operon of *S. typhimurium* [49]. In case of transcription inhibition and for high cobalamin concentrations, an alternative Rho-independent termination hairpin or Rho binding site cause premature transcriptional termination. High cobalamin concentrations can also promote sequestration of ribosome binding sites (RBS) and blockage of translation initiation. When cobalamin concentrations are low, an anti-terminator hairpin forms, enabling RNA polymerase to complete transcription of the downstream gene. Low cobalamin concentrations facilitate anti-sequester hairpin formation that releases the RBS, thus enabling translation initiation [50, 51]. Cobalamin riboswitches also have a particular function in ethanolamine utilization. In *Listeria monocytogenes*, a cobalamin riboswitch controls the expression of a noncoding regulatory RNA named Rli55, which controls the expression of the *eut* genes that requires vitamin B_12_ as a cofactor and determines ethanolamine utilization [52].

## Synthetic biology to improve cobalamin production

### Design of a heterologous biosynthetic pathway for the vitamin B_12_ production

**Fig. 2:**
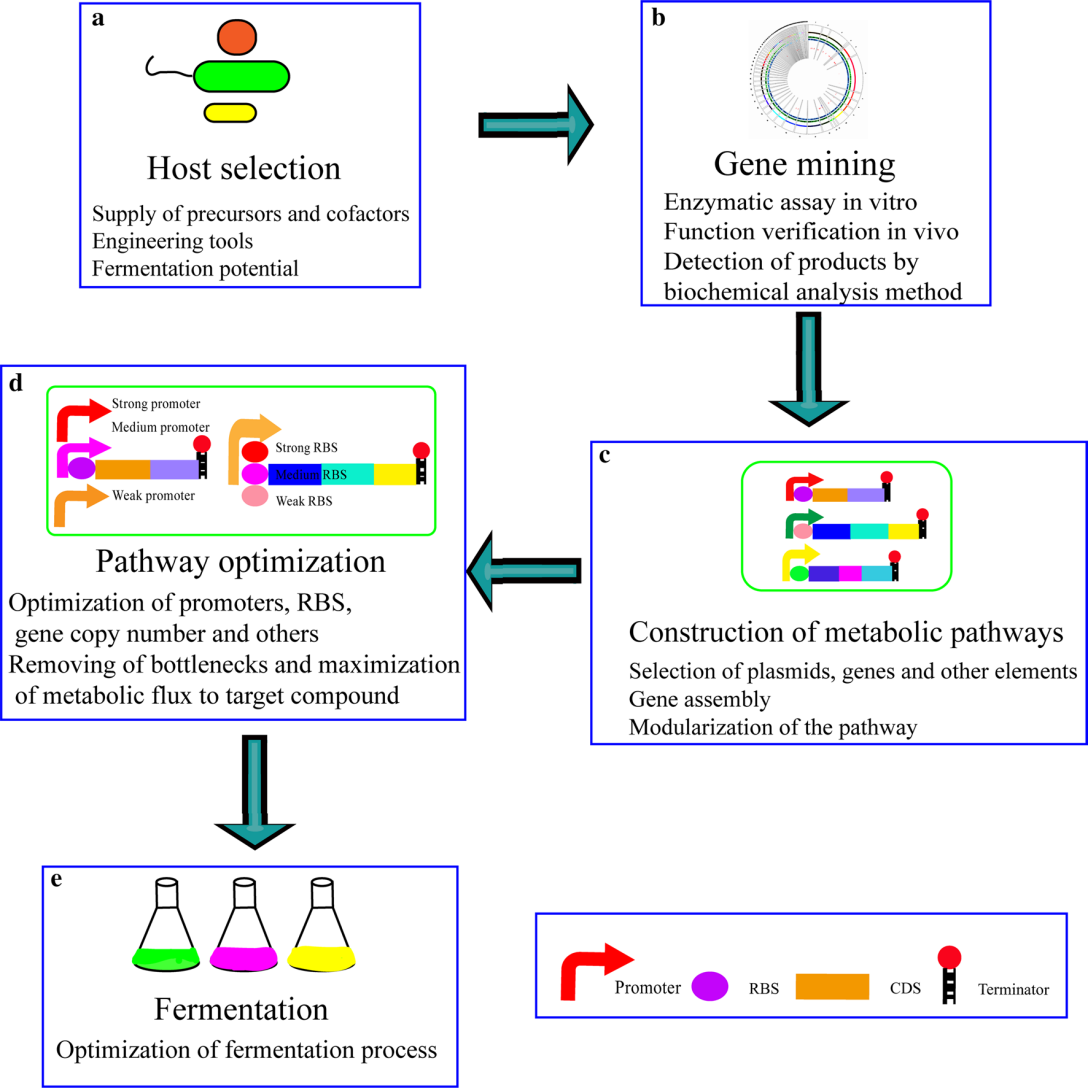
Design of a heterologous biosynthetic pathway. **a** A host for the heterologous biosynthetic pathway is selected considering the capability of precursor and cofactor supply, genetic engineering tools, and industrial-scale fermentation capability, utilizing cheap and readily available carbon sources. **b** Enzyme activity is verified in vitro and subsequently in vivo. Products of the in vitro assay or intracellular reaction products are detected via spectroscopic analysis, mass spectrometry, or microbiological assays. **c** Heterogeneous genes and other functional elements are assembled on plasmids via gene assembly methods such as SLIC, CPEC, Gibson, golden gate, DNA assembler and LCR, or integrated into the genome. To decrease the difficulty of building the metabolic pathway, it is divided into separate modules. These modules are verified sequentially in a heterologous host and then assembled. **d** Based on the quantification of metabolites, bottlenecks should be removed and metabolic flux should be integrated to target compound maximization. To optimize gene expression in the metabolic pathway, promoters, RBS, and gene copy number are designed and implemented at the transcriptional or translational levels. **e** The characteristics of the engineered strains are verified via fermentation. Various substrates (e.g., ALA, cobalt ions, betaine and DMB) and varying conditions (e.g., dissolved oxygen concentration, pH, and temperature) can be optimized to improve yield and productivity

Apart from altering native microbial hosts or identifying novel microbial hosts to produce cobalamin, the construction of heterologous biosynthetic pathways in model organisms that can be easily genetically manipulated is a promising strategy. Synthetic biology is an efficient tool that can be used to reconstruct pathways or genetic networks to produce compounds in a heterologous host. The construction of a vitamin B_12_ biosynthetic pathway in a heterologous host includes selection of a suitable host, building the biosynthetic pathway with functional components, and pathway tuning (Fig. 2). Several points should be noted in the selection of an ideal host. (1) The host should have the ability to supply precursors (e.g. ALA) and cofactors (e.g. S-adenosylmethionine) for the production of the desired chemical. E.g., the heterologous C_4_ pathway in *E. coli* has been reported to generate high ALA production [13], avoiding addition of exogenous ALA to the medium; (2) there need to be sufficient genetic engineering tools such as transformation protocols, expression vectors, and chromosomal gene knockout/integration systems to manipulate the host [53]; (3) the host is suitable for industrial-scale fermentation, utilizing cheap and readily available carbon sources such as glucose, xylose, and arabinose. When the host is selected, various candidate genes from diverse native vitamin B_12_ producers can be expressed in the host. The use of in vitro kinetic analysis is an efficient approach to detect ideal enzymes. Most of the intermediates of the vitamin B_12_ synthetic pathway are not available; consequently, to prepare substrates for in vitro kinetic analysis, the desired chemicals need to be separated from the products of corresponding strains or be prepared enzymatically. The products of an in vitro assay can then be detected by spectroscopic analysis, mass spectrometry, or microbiological assays. Sometimes, the heterologous production of enzymes does not work and it is necessary to screen for novel enzymes from various sources. For heterologous expression studies, all native forms of regulation, such as riboswitches, should be removed. After the addition of transcriptional and translational elements, such as promoters, ribosome binding sequences, and terminators, the structural genes for cobalamin synthesis can be expressed either monocistronically or polycistronically on plasmids or be integrated into the genome. The rapid development of synthetic biology tools has facilitated the simplified construction of heterologous pathways. Heterogeneous genes can be assembled by a number of different techniques such as SLIC, CPEC, Gibson assembly, golden gate cloning, DNA assembler, and LCR [54]. When too many heterogeneous genes need to be transferred to a heterologous host, it is difficult to build the metabolic pathway one gene at a time. The metabolic route can be split into multiple modules. After sequential validation of the function of these modules, their assembly becomes possible. The final construct can then be transferred to the chosen host for heterologous expression, thus allowing the host to synthesize cobalamin. To evaluate the capacity of vitamin B_12_ production, the engineered strains are then cultured under optimal conditions.

Design of a heterologous biosynthetic pathway. (A) A host for the heterologous biosynthetic pathway is selected considering capabilities of precursor and cofactor supply, genetic engineering tools, and industrial-scale fermentation capability using cheap and readily available carbon sources. (B) Enzyme activity is verified in vitro and thereafter in vivo. Products of in vitro assay or intracellular reaction products are detected via spectroscopic analysis, mass spectrometry, or microbiological assays. (C) Heterogeneous genes and other functional elements are assembled on plasmids via gene assembly methods such as SLIC, CPEC, Gibson, golden gate, DNA assembler, and LCR, or integrated into the genome. To decrease the difficulty of building the metabolic pathway, it is divided into separate modules. These modules are sequentially verified in a heterologous host, and then assembled. (D) Based on the quantification of metabolites, bottlenecks should be removed and metabolic flux to target compound maximization. To optimize gene expression in the metabolic pathway, promoters, RBS, and gene copy number are designed and implemented at the transcriptional or translational levels. (E) The characteristics of engineered strains are verified via fermentation. Various substrates (e.g., ALA, cobalt ions, betaine and DMB) and varying conditions (e.g., dissolved oxygen concentration, pH, and temperature) can be optimized to improve yield and productivity.

### Pathway tuning

Biologists face the challenge to direct the flux of intermediates to generate the desired product. To avoid the accumulation of toxic intermediates and to drive flux to the desired end product, each step in the pathway needs to be balanced. In addition, gene overexpression may cause an undesirable metabolic burden on the host, and the level of gene expression needs to be accurately adjusted to coordinate both metabolic flux and cell growth. Sometimes chromosomal expression of heterologous metabolic pathways can avoid plasmid instability and thus reduce the metabolic burden on the cell. Vitamin B_12_ biosynthesis is a highly evolved pathway, with more than twenty steps, including corrinoid ring construction and peripheral modifications, and the enzymes involved have high substrate specificity. Therefore, it is necessary to optimize gene expression in existing metabolic pathways.

Gene expression in a given pathway can be altered at the level of transcription or translation [55]. Promoter strength affects gene expression at the transcriptional level, while DNA sequence of the RBS affects gene expression at the translational level. The “Ribosome binding site calculator” and RBSDesigner have been used to predict and design synthetic RBSs to yield the desired level of protein expression in bacteria [56]. The expression of multiple genes in synthetic operons can be optimized to enhance production levels via engineering of promoters [57] and RBSs [58], or via generation of libraries of tunable intergenic regions [59]. Tuning operon expression can be achieved via different plasmid replicons to control gene copy number, different promoters to control the rate of transcription initiation, and different RBSs for controlling the level of translation [60]. Multiplex automated genome engineering (MAGE) can be used for genome-scale optimization of gene expression on the chromosome [61].

The translational efficiency of each gene in an operon is affected by intergenic sequence regions and involves post-transcriptional processes such as transcription termination, mRNA stability, and translation initiation. Therefore, achieving the desired expression level of each gene within an operon remains challenging. Pfleger et al. generated libraries of tunable intergenic regions, recombining various post-transcriptional control elements to optimize the expression of multiple genes in a synthetic operon [62]. Tian et al. also designed synthetic operons that utilize translational coupling to achieve the desired expression level ratios [63].

Scaffolding is a way to rapidly increase overall pathway flux and is complementary to these conventional methods. Scaffolding can increase the effective concentration of intermediates in the pathway of interest, via the recruitment of enzymes to synthetic protein scaffolds. Three mevalonate biosynthetic enzymes (acetoacetyl-CoA thiolase, hydroxy-methylglutaryl-CoA synthase, and hydroxymethylglutaryl-CoA reductase) were recruited to a synthetic protein scaffold through interactions between the GTPase binding domain, the SH3 domain, the PSD95/DlgA/Zo-1 domain and their respective ligands, increasing mevalonate yields 77-fold compared to cells without the scaffold [64]. Protein scaffolds simulate natural multienzyme complexes and have been used to solve problems of toxic pathway intermediate accumulation, competing metabolic reactions, and imbalances in flux [65]. Protein scaffolds have been successfully applied in various other pathways, including the glucaric acid pathway, where three pathway enzymes were joined in a protein scaffold, increasing titers by approximately five-fold [66]. In another example, heterologous butyrate pathway enzymes were co-localized in a protein scaffold, resulting in a threefold improvement in butyrate production [67]. Cobalamin synthesis is a complex pathway with several competing intermediates, such as heme and siroheme. Co-localization of enzymes to the same subcellular organelle or compartment can increase the local intermediate concentration and exclude competing cytosolic pathways [68]. Substrate channeling is a process of transferring the product of one enzyme to an adjacent enzyme within the cascade or cell without complete mixing with the bulk phase [69]. This phenomenon occurs for at least some of the enzymes involved in hydrogenobyrinic acid (HBA) biosynthesis. An enzyme-trap approach has been used to isolate intermediates of the cobalamin biosynthetic pathway based on this mechanism [70]. Therefore, protein scaffolds may offer a promising alternative to balance multiple enzymes within the vitamin B_12_ biosynthetic pathway, thus improving flux to cobalamin.

All these approaches optimize gene expression or increase pathway flux in vivo. In vitro steady-state analysis is an efficient approach to identify bottlenecks in metabolic pathways and to balance flux to the desired compounds. It has been successfully used to increase the production of fatty acids, fatty acid short-chain esters, fatty alcohols, farnesenes, alkenes, and alkanes [71–75]. Previously, we have optimized the concentration of enzymes involved in precorrin-2 synthesis in vitro via response surface methodology [76]. This approach minimizes the effects of substrate inhibition and feedback inhibition by SUMT and increases the production of precorrin-2 approximately five-fold.

### Construction of a heterologous biosynthetic pathway for cobalamin in *E. coli*

*Escherichia coli* is a well-characterized prokaryote that has been used as a microbial cell factory for many chemicals. Although it has lost the de novo cobalamin synthesis pathway during evolution, *E. coli* can synthesize cobalamin through the salvage pathway, thus saving resources and energy. *Escherichia coli* has been used to produce ALA in many studies. A sufficient ALA supply is necessary for the vitamin B_12_ biosynthesis. This implies that *E. coli* may be a suitable host for vitamin B_12_ production. Other common bacteria such as *Corynebacterium glutamicum* and *Bacillus subtilis* lack the genes involved in the cobalamin synthesis pathway after precorrin-2, which may explain why the construction of a heterologous de novo cobalamin synthesis pathway in bacteria other than *E. coli* has yet to be reported.

For a long time, cobalamin production was restricted to native bacterial producers, with the exception of several studies that focused on the determination of gene function in the cobalamin synthesis pathway in vivo [16, 35, 70, 77] or by using cell extracts from recombinant *E. coli* in vitro (Table 2) [78–82]. After insertion of the *B. megaterium cob* I operon and *S. typhimurium cbiP*, *E. coli* was able to synthesize cobyric acid de novo [77]. Co-expression of the *cobA* gene from *Propionibacterium freudenreichii* and the *cbiAP, cbiCDETFGHJ* and *cbiJKL* genes from *S. typhimurium* equipped *E. coli* with the ability to produce cobyrinic acid a,c-diamide [16]. Apart from the genes involved in the anaerobic cobalamin biosynthetic pathway, genes involved in aerobic cobalamin biosynthetic pathway have also been shown to work in *E. coli*. Furthermore, *E. coli* possesses enzymes that perform the transformation of uroporphyrinogen III into HBA, thus potentially producing HBA [70]. McGoldrick et al. conducted a similar experiment, where nine genes from *R. capsulatus* and *Brucella melitensis*, encoding enzymes for the transformation of uroporphyrinogen III into HBA, were constructed as an operon in pET14b and introduced into *E. coli*, allowing the host to produce HBA [35]. To the best of our knowledge, there are only two reports on de novo cobalamin synthesis in *E. coli*: Firstly, an *E. coli* strain harboring a plasmid with a 21.5-kb native operon (*pduBAF*-*pocR*-*cbiABCDETFGHJKLMNQOP*-*cobUST*) from *S. typhimurium* resulted in de novo cobalamin biosynthesis [18]. Recently, Ko et al. also accomplished cobalamin biosynthesis in *E. coli* [83]. Twenty-two genes located in six different operons of *P. denitrificans* were cloned via traditional restriction and ligation into three compatible plasmids under the control of a T7 promoter. The recombinant *E. coli* strain harboring these three plasmids produced vitamin B_12_ under both anaerobic and aerobic conditions. Via optimizing the culture conditions, the engineered strain produced 0.65 ± 0.03 μg/g cdw of coenzyme B_12_. These examples show that *E. coli* can be utilized to produce vitamin B_12_ or pathway intermediates de novo through the aerobic or anaerobic pathway.

**Table 2:** Research on the biosynthesis of vitamin B_12_ and its intermediates in vivo and in vitro in *E. coli*

Strains	Products	Strategies	References
In vivo			
*E. coli*	Cobyric acid	Expression of the *cob* I operon of *B. megaterium* and *cbiP* of *S. typhimurium*	[77]
*E. coli*	Cobyrinic acid a,c-diamide	Co-expression of the *cobA* gene from *P. freudenreichii* and the *cbiAP, cbiCDETFGHJ*, and *cbiJKL* genes from *S. typhimurium*	[16]
*E. coli*	HBA	Co-expression of *cobA*, *cobI*, *cobJ*, *cobF, cobM*, *cobK*, *cobL*, and *cobH* from *R. capsulatus* SB1003, and *cobG* from *B. melitensis* 16M or *P. denitrificans*	[70]
*E. coli*	HBA	Co-expression of *cobA*, *cobI*, *cobJ*, *cobF*, *cobM*, *cobK*, *cobL*, *cobE*, *cobH* and *cobZ* from *R. capsulatus*	[35]
*E. coli*	Vitamin B_12_	Expression of the operon *pduBAF*-*pocR*-*cbiABCDETFGHJKLMNQOP*-*cobUST* from *S. typhimurium*	[18]
*E. coli*	Vitamin B_12_	Co-expression of *cobWNEMcbtAB, tonBcobOBRDCQchlID* and *cobGHIJLFK* from *P. denitrificans*	[83]
In vitro			
*E. coli*	HBA	*E. coli* native genes *hemB*, *hemC*, *hemD*, and *cobA*, *cobI*, *cobG*, *cobJ*, *cobF*, *cobM*, *cobK*, *cobL*, and *cobH* genes from *P. denitrificans* were heterologously expressed in *E. coli*. HBA was produced when ALA was added to the media and the cells were incubated aerobically in 200 ml Tris–HCl buffer, pH 8.0, containing SAM, NADH, and NADPH for 15 h at 30 °C with a mixture of twelve enzymes: HemB, HemC, HemD, crude enzyme extract of CobA, CobI, CobG, CobJ, CobM, CobF, CobK, CobL, and CobH	[78]
*E. coli*	Precorrin3b and precorrin-4	The *cobG* and *cobJ* genes from *P. denitrificans* were heterologously expressed in *E. coli*. Aerobic incubation of precorrin-3A with the CobG enzyme from *P.* denitrificans alone yielded precorrin3B. When CobJ from *P.* denitrificans and S-adenosyl-l-methionine were included in the reaction, the product precorrin-4 was formed	[18]
*E. coli*	Cob(II)yrinic acid a,c-diamide	The *cobN*, *cobS*, and *cobT* from *B. melitensis* were expressed and purified from *E. coli*. In the presence of ATP, Co^2+^, and hydrogenobyrinic acid a,c diamide, these enzymes together produced cobyrinic acid	[81]
*E. coli*	Cob(I)yrinic acid a,c-diamide	*CobR* from *B. melitensis* was expressed and purified from *E. coli*. This enzmye was confirmed to have co(II)rrin reductase activity	[82]

## Metabolic engineering for cobalamin production

### Scheme of metabolic engineering

When a micro-organism possesses its own or a heterologous cobalamin synthesis pathway, efforts should be directed towards engineering the metabolic network to enhance vitamin B_12_ production and yield. Classical metabolic engineering involves an iterative process of synthesis and analysis, where increasingly refined strains are designed and constructed based on gathered knowledge [84]. However, systems metabolic engineering allows microbes to be engineered at the whole-organism level for the production of valuable chemicals far beyond their native capabilities [85]. Metabolic design based on in silico simulations and experimental validation of the metabolic state in the engineered strain facilitates systematic metabolic engineering [86]. Many genome-scale metabolic flux models have been developed to design microbial cell factories. In silico simulations based on genome-scale metabolic models have provided valid guidance for rational design. Computational tools used in metabolic flux analysis and gene manipulation studies have been systematically reviewed [87]. Depending on the design of the specific strain, experimental implementation can involve a combination of gene over-expression, introduction of foreign enzymes, gene deletions, or knockdowns and the modification of enzyme properties.

### Gene over-expression and heterologous expression

Gene over-expression is used to enhance or redirect flux to the desired chemical reaction. Expression of the target gene can be increased through the use of multi-copy plasmids, or the insertion of transcriptional and translational control elements, such as strong promoters, highly efficient RBSs, and terminator sequences. The introduction of heterologous genes is used to overcome the comparatively low activity of native genes in a host. The structural genes required for cobalamin synthesis are typically targets for gene over-expression to boost cobalamin production. In a recombinant *P. freudenreichii* strain, which harbors an expression vector containing *cobA*, *cbiLF*, or *cbiEGH*, vitamin B_12_ production was increased 1.7-, 1.9-, and 1.5-fold, respectively, compared to *P. freudenreichii* harboring the expression vector pPK705 [88]. Expression of *cobU* and *cobS* in the same *P. freudenreichii* strain led to a slight increase in the production of vitamin B_12_. ALA is a precursor, restricting cobalamin synthesis and a direct strategy to overcome this is to up-regulate the availability of ALA. A recombinant *P. freudenreichii* strain with heterologous *hemA* from *R. sphaeroides*, *hemB*, and *cobA* homologues, revealed a 2.2-fold increase in vitamin B_12_ production [88]. To improve the production of tetrapyrrole compounds, the *hemA* gene from *R. sphaeroides* and the *hemB* gene from *P. freudenreichii* subsp. *shermanii* IFO12424 were expressed either monocistronically or polycistronically in the strain *P. freudenreichii* subsp. *shermanii* IFO12426. The recombinant strains accumulated larger amounts of ALA and PBG, with a resultant 28- to 33-fold increase in the production of porphyrinogens, compared to strain *P. freudenreichii* subsp. *shermanii* IFO12426, harboring the vector pPK705 [89]. A cobalamin-riboswitch can be used to inhibit excess vitamin B_12_ in microbes. To bypass the effects of the cobalamin-riboswitch, a *cbi* operon without the cobalamin-riboswitch was cloned. Growth of *B. megaterium*, harboring a plasmid expressing the *cbi* operon on minimal media supplemented with glycerol as a carbon source, resulted in a significant increase in cobalamin production (up to 200 μg/l) [90]. All these strategies focus on the over-expression of structural genes that are directly involved in the vitamin B_12_ biosynthesis. However, to the best of our knowledge, no published reports exist on other genes, such as those responsible for the S-adenosylmethionine (SAM) or DMB metabolism. This suggests that research into the metabolic engineering of vitamin B_12_ production is still in its infancy.

### Inactivation or down-regulation of genes

A further general method of metabolic engineering is to knockout genes to improve precursor supply, or to eliminate by-products or competing chemical synthesis reactions. Genes that encode enzymes at specific branch points in the metabolic pathway are good targets for this strategy. For essential genes that cannot be deleted, clustered regularly interspaced short palindromic repeats (CRISPR), CRISPR interference (CRISPRi), sRNA, or regulatory RNA can be used to repress the desired genes [91, 92]. Many genome editing tools such as, traditional homologous recombination systems and ZFN (zinc finger nucleases), TALEN (transcription activator-like effector nucleases), and CRISPR/Cas-based methods readily enable genetic modifications. Heme and siroheme are competitive byproducts of the vitamin B_12_ synthesis. To reduce flux to the heme branch of the tetrapyrrole pathway, antisense RNA was used to silence *hemZ* (which encodes coproporphyrinogen III oxidase) in *B. megaterium*. This approach led to a 20% increase in the intracellular vitamin B_12_ concentration [93]. We also inhibited *hemE* and *hemH* to improve flux to precorrin-2 via an sRNA approach (unpublished data).

### Protein engineering

During the engineering of native or heterologous hosts to produce a desired chemical, a common scenario is that a substance or product inhibits an enzyme or it may not function heterologously. Protein engineering is a useful approach to improve the specificity, solubility, and stability of enzymes. It includes directed evolution, semi rational design, and rational design. The enzyme glutamyl-tRNA reductase (HemA) catalyzes the first committed step in the heme biosynthetic pathway. Expression of this gene is inhibited in a negative feedback loop by excess heme. The addition of two positively charged lysine residues to the third and fourth positions of the N terminus of HemA resulted in a complete stabilization of the protein. Cells expressing the stabilized HemA showed a substantial decrease in heme inhibition [94]. Engineering of CobA to remove substrate inhibition or the identification of novel genes without substrate inhibition may improve the yield of vitamin B_12_.

### ^13^C-metabolic flux analysis

^13^C-metabolic flux analysis is an efficient method to determine flux distribution based on experimental data and has been used to accurately estimate flux in the central carbon metabolism of *P. denitrificans* in response to different specific oxygen uptake rates under oxygen limiting conditions [95]. Metabolic flux analysis revealed that glucose was predominantly catabolized by the Entner–Doudoroff and pentose phosphate pathways. A higher specific oxygen uptake rate accelerated the supply of precursors, methyl groups, and NADPH to increase vitamin B_12_ production [95].

## Other strategies to improve cobalamin production

### Random mutagenesis

To produce strains with a high cobalamin yield, a conventional strategy is random mutagenesis. Specifically, UV light or chemical mutagens can both be used to treat the respective microorganisms and then, strains with the desired phenotype, such as improved productivity, genetic stability, reasonable growth rates, or resistance to high concentrations of toxic intermediates can be selected [7]. High throughput screening methods based on signals, such as survival and fluorescence, have been used to obtain desired mutants from large libraries. Moreover, random mutagenesis can also be applied to inverse metabolic engineering for targeted transformation.

### Genome-scale engineering

Genome shuffling, an approach that combines random mutagenesis and protoplast fusion, has been used to improve vitamin B_12_ production in *P. shermanii*. The engineered *P. shermanii* strain produced approximately 61% more vitamin B_12_ than the parent strain after 96 h. Comparative proteome analysis revealed that the expression of 38 proteins varied significantly [96]. Compared to genome shuffling, which results in random mutations, MAGE provides an efficient method to simultaneously modify multiple genomic locations [61]. MAGE is based on the λ red recombination system. The repetitive introduction of ssDNA targeting multiple loci in the *E. coli* genome results in various mutants. Combined with standard high-throughput screening methods, MAGE may represent a rapid and efficient tool to obtain “ideal” bacterial producers. In addition, another versatile method, trackable multiplex recombineering (TRMR), enables rapid and simultaneous modification and mapping of thousands of genes. By changing functional regions such as promoters, translation sites, switches, oscillators, or sensors, a broad range of studies can be performed [97].

### Biosensors

Biosensors have been extensively applied in high-throughput assays. For chemicals that cannot be easily detected via measurement of absorption or fluorescence, biosensors indirectly reflect chemical concentrations. A vitamin B_12_ riboswitch sensor has been used to study synthesis and import of coenzyme B_12_, and was shown to detect intracellular vitamin B_12_ concentration using colorimetric, fluorescent, or luminescent reporters with high sensitivity [98]. Reporter genes integrated with the *btuB* riboswitch have been used to monitor intracellular AdoCbl concentrations [98]. A combination of evolutionary strategies applied to metabolic pathways, whole genomes, or biosensors may be a useful approach to advance high-throughput screening for “ideal” strains.

### Fermentation process optimization

The addition of precursors of the vitamin B_12_ biosynthetic pathway, such as cobalt ions, ALA, DMB, glycine, threonine, or compatible solutes like betaine and choline has been frequently described to be beneficial [7]. Propionic acid is a byproduct of the vitamin B_12_ cultivation process in *P. freudenreichii* and causes feedback inhibition of microbial cell growth. Propionic acid production and DMB addition were controlled via expanded bed adsorption bioreactors, which were shown to improve vitamin B_12_ biosynthesis [99]. Mixed culture of *Propionibacterium* and *Ralstonia eutropha* have also been used to solve this problem, as the latter can assimilate propionic acid produced by the former [100]. Betaine is an important methyl-group donor for the formation of methionine, which is further converted to SAM by the activity of methionine adenosyltransferase. S-adenosylmethionine is an important precursor during corrinoid ring formation. Despite the fact that betaine delays cell growth, betaine feeding during fermentation has been revealed as an effective strategy to increase vitamin B_12_ production [101]. To reduce medium and fermentation costs, cheap carbon sources such as maltose syrup and corn steep liquor can be used to replace refined sucrose [102].

## Conclusions

Vitamin B_12_ is widely used in medical and food industries and microbes produce vitamin B_12_ via an intricate pathway. Tetrapyrrole compounds, including heme, cobalamin, and siroheme are not simple competitive compounds, but have both interdependent and interactive relationships in several bacterial species. To maintain stable vitamin B_12_ concentrations, its biosynthesis and transport are regulated at the transcriptional or translational level via riboswitches. Vitamin B_12_ is produced by microbial fermentation, using strains such as *P. denitrificans* and *P. shermanii*. Since relatively few genetic tools are available for these strains, and the fermentation process is complicated, strain engineering has focused on traditional strategies such as random mutagenesis and the optimization of the fermentation process. It is imperative to introduce new engineering tools, such as systems metabolic engineering, to manipulate these strains. Apart from native producers of vitamin B_12_, *E. coli* has also been used as a heterologous host. To provide guidance for the construction of microbial cell factories for vitamin B_12_ production, we summarized synthetic biology and metabolic engineering strategies, as well as other traditional strategies that either have been or could be applied to vitamin B_12_ production. These strategies have been extensively applied in microbial strain engineering to improve the production of many other chemicals. Based on a clear understanding of the vitamin B_12_ metabolism in microbes, the utilization of these strategies should promote an improved microbial vitamin B_12_ production.

## Abbreviations

ALA: δ-aminolevulinate
UTR: untranslated regions
ACAT: ATP co(I)rrinoid adenosyltransferase
AdoCbi: adenosylcobinamide
AdoCbl: adenosylcobalamin
NADH: reduced form of nicotinamide-adenine dinucleotide
FMN: flavin mononucleotide
NADPH: reduced nicotinamide adenine dinucleotide phosphate
ABC: ATP-binding cassette
SUMT: S-Adenosyl-l-methionine:uroporphyrinogen III methyltransferase
HBA: hydrogenobyrinic acid
RBS: ribosome-binding site
MAGE: multiplex automated genome engineering
CRISPR: clustered regularly interspaced short palindromic repeats
CRISPRi: CRISPR interference
TRMR: trackable multiplex recombineering
ZFN: zinc finger nucleases
TALEN: transcription activator-like effector nucleases
